# Sexual health at the end of life in patients with advanced cancer and their partners. Results of a Dutch prospective longitudinal study (eQuiPe)

**DOI:** 10.1177/02692163251385774

**Published:** 2025-11-14

**Authors:** Isabel S. van der Meer, Moyke A. J. Versluis, Heidi P. Fransen, Corien M. Eeltink, Arnold Baars, Dirkje W. Sommeijer, Tineke J. Smilde, Annemieke van der Padt-Pruijsten, Lonneke V. van de Poll-Franse, Natasja J. H. Raijmakers

**Affiliations:** 1 Department of Research and Development, The Netherlands Comprehensive Cancer Organisation, Utrecht, The Netherlands; 2 Graduate School of Social and Behavioral Sciences, Tilburg University, The Netherlands; 3 Department of Gastrointestinal Cancer, Dijklander Hospital, Purmerend/Hoorn, The Netherlands; 4 Department of Internal Medicine, Hospital Gelderse Vallei, Ede, The Netherlands; 5 Department of Medical Oncolgy, Amsterdam University Medical Centre, Amsterdam, The Netherlands; 6 Department of Medical Oncology, Flevo Hospital, Almere, The Netherlands; 7 Department of Medical Oncology, Jeroen Bosch Hospital, ’s-Hertogenbosch, The Netherlands; 8 Department of Internal Medicine, Maasstad Hospital, Rotterdam, The Netherlands; 9 Division of Psychosocial Research and Epidemiology, The Netherlands Cancer Institute, Amsterdam, The Netherlands; 10 CoRPS – Center of Research on Psychological Disorders and Somatic Diseases, Departmentc of Medical and Clinical Psychology, Tilburg University, The Netherlands

**Keywords:** sexual health, sexual partners, longitudinal studies, palliative care, quality of life

## Abstract

**Background:**

Sexual health can be negatively affected by cancer, yet little is known about changes at the end of life.

**Aim:**

To assess changes in sexual health in patients with advanced cancer and their partners at the end of life, and identify associated factors.

**Design:**

Prospective longitudinal study of patients with advanced cancer and their partners on quality of life and care (eQuiPe). Patients and partners completed three-monthly questionnaires until patient’s death. Sexual health was measured in all questionnaires using the EORTC QLQ-SH22, covering four subdomains (desire, activity, satisfaction and enjoyment; scores 0–100).

**Setting/participants:**

Patients, aged ⩾ 18 years, diagnosed with stage IV cancer were recruited from 40 Dutch hospitals. Relatives were recruited through patients. For this study only couples (patient-partner) were included (*n* = 352).

**Results:**

Median number of completed questionnaires per couple was one (range 1–7). Toward death, patients remained relatively stable in sexual activity (range 15–19), satisfaction (range 39–45), and enjoyment (range 30–45). Similar results were found for partners. In patients, sexual desire significantly decreased toward death (β 0.4, 95% CI 0.1–0.7). Greater decline in physical functioning and being diagnosed with prostate cancer was associated with poorer outcomes in most sexual health domains. Sexual desire, activity, and satisfaction were individually associated with quality of life in patients.

**Conclusions:**

Sexual health remains relatively stable at the end of life in patients with advanced cancer and their partner. Patients with worse physical functioning and/or prostate cancer report worse sexual health. Sexual desire, activity, and satisfaction are individually associated with better quality of life.

**What was already known?**

- The diagnosis of advanced cancer and subsequent treatments can have negative implications for sexual health
- Changes in sexual health of patients with advanced cancer emerge from physical, mental and emotional transformations, but the importance of sexual health remains relatively unchanged.
- The majority of healthcare professionals find it challenging to discuss sexual health in the context of palliative care.

**What this paper adds?**

- Patients and their partner remain relatively stable in most aspects of sexual health in the last 18 months of the patients’ life.
- Patients’ sexual desire significantly decreases in their last 18 months of life.
- Patients with worse physical functioning and/or prostate cancer reported a greater decline in most aspects of sexual health.
- Patients’ sexual desire, activity and satisfaction were individually associated with the quality of life in the last 18 months of life.

**Implications for practice, theory, policy, or future research?**

- Recognizing sexual health as an integral component of overall quality of life is essential.
- Discussing sexual health as healthcare professionals is important. Using short PROM’s exploring the patient’s need to discuss sexual health could facilitate the initiation of such a discussion.
- Future research is essential to examine whether patients perceive decreased sexual health as a concern and whether the meaning of sex changes at the end-of-life.

## Background

Cancer remains one of the leading causes of death worldwide, with approximately 20 million new cases diagnosed in 2022.^1^ Despite significant medical advances in oncological care, patients with advanced cancer still have a limited median survival.^2,3^ The diagnosis and treatment of advanced cancer have a significant impact on patients and their relatives, and maintaining or improving the quality of life for these patients is paramount.

Sexual health is an integral part of quality of life and consists of several components, including sexual activity, enjoyment, satisfaction, and desire.^4^ Advanced, incurable cancer and subsequent treatments can have negative implications for sexual health, both for patients as their partners.^5^ Over 40% of patients with curable cancer encounter sexual problems post-treatment.^6,7^ The majority of patients with advanced cancer (75%) reported low sexual satisfaction and lower levels of sexual activity.^8,9^ Moreover, patients with advanced cancer reported lower sexual health compared to patients with curable cancer, due to side effects of treatment and physical changes at the end of life.^10^

Sexual health may change significantly throughout the final phase of life, as patients with advanced cancer experience various physical, mental, and emotional transformations. These include an increased symptom burden and a decreased quality of life for patients and a decline in emotional well-being for their relatives.^11,12^ Additionally, as patients become more reliant on their partners for care, the relationship dynamics may shift, altering social roles and potentially impacting sexual health and intimacy.^13,14^ Despite these changes, the importance of sexuality for patients with advanced cancer remains relatively unchanged at the end of life.^8,15,16^

Current literature consists mainly of cross-sectional studies on sexual health, with a limited understanding of the changes in sexuality during at the end of life.^9,15–17^ Additionally, the majority of sexual health studies have focused on sexual functioning^7,10,18–21^ and on cancer survivors,^14,20,22–24^ with an emphasis on female patients.^19,24–29^ Understanding how sexual health changes in the end of life is needed to support patients and their partners to optimize quality of life. Such knowledge is essential to raise awareness among healthcare providers of the importance of sexual health in the management of advanced cancer. Therefore, the aim of this study was to assess the changes in sexual health among adult patients with advanced cancer and their partners in the end of life, and the factors associated with these changes.

## Methods

### Study design

A longitudinal, multicenter, prospective, observational cohort study (eQuiPe) was conducted to gain insight in the experienced health-related quality of life and quality of care of patients with advanced cancer and their relatives in the Netherlands.^30^

### Study population

All patients aged ⩾ 18 years, diagnosed with stage IV cancer, and able to understand and complete a Dutch questionnaire deemed eligible for inclusion. To prevent the inclusion of patients with a relatively good prognosis, additional criteria were defined for patients with breast or prostate cancer. Patients with breast cancer were eligible when their cancer was metastasized to multiple organs and patients with prostate cancer were included when their cancer was metastasized and castrate resistant. Relatives were eligible when aged ⩾ 18 years and when they were able to complete a Dutch questionnaire. Both patients and relatives were excluded if diagnosed with dementia or if they had a history of severe mental illness.

### Sampling

The eQuiPe study is a nationwide study that was conducted in 40 Dutch hospitals at the departments of medical oncology, pulmonology and/or urology. Eligible patients were either identified by their treating physician in one of the participating hospitals or were self-registered via an advertisement on a Dutch platform for patients and relatives who are confronted with cancer (www.kanker.nl) between November 2017 and March 2020. Relatives were asked to participate by the patient.

### Recruitment

Patients and relatives were contacted by phone by the research team to check eligibility and to explain the details of the eQuiPe study. After confirming participation, patients and relatives were asked to sign a written informed consent, either on paper or online.

### Data collection

When written informed consent was signed, patients and relatives received a baseline questionnaire followed by subsequent three-monthly follow-up questionnaires until the patient’s death. Questionnaires were administered either on paper or online via the Patient Reported Outcomes Following Initial treatment and Long-term Evaluation of Survivorship (PROFILES).^31^ Clinical data of patients were obtained by linking to the to the Netherlands cancer registry.^32^

### Ethical considerations

The eQuiPe study was exempted from full medical ethical review by the Antoni van Leeuwenhoek’s hospital Medical Ethics Committee (METC17.1491), in accordance with the Dutch Medical Research Involving Human Subjects Act (WMO). Registration details of the study can be accessed through the Netherlands Trial Register under the identifier NL6408.

All participants provided written informed consent prior to inclusion. Participation was voluntary, and participants could withdraw at any time without consequences. Given the vulnerability of the population, a pilot study was conducted prior to the main eQuiPe study, which showed that the most extensive questionnaire had a completion time of 38 min and was not experienced as burdensome, confrontational, incomprehensible or inappropriate.^30^

### Measures

#### Sexual health

Sexual health was assessed at baseline and three-monthly follow-up using the European Organization for Research and Treatment of Cancer Quality of Life Questionnaire Sexual Health (EORTC QLQ-SH22).^33,34^ The subscales comprised four questions related to the past 4 weeks, measuring sexual desire, sexual activity, sexual satisfaction, and sexual enjoyment. Response options included a 4-point Likert scale ranging from 1 ‘Not at all’ to 4 ‘Very much’. Responses were linearly transformed to a 0–100 scale, with higher scores indicating better outcomes.

#### Socio-demographic and clinical characteristics

Age, sex, religion, education level, relationship duration, having children, and children living at home were self-reported at baseline. Education was categorized according to the International Standard Classification of Educational Guidelines: low, middle and high.^35^ Patients and partners were asked about their religious affiliation, with response options including specific religions as well as ‘not religious’. Due to the low diversity in religious affiliations, we grouped all religious responses into a single category, ‘being religious’ and compared this group to those who identified as ‘not religious’. Comorbidities were measured at baseline using the Self-administered Comorbidity Questionnaire (SCQ).^36^ Primary tumor type and date of death were obtained from the Netherlands cancer registry.^32^ Time to death was defined as the time (in months) between completion of the questionnaire and date of death. Subsequently, in each questionnaire, patients reported if they received cancer treatment (including chemotherapy, radiotherapy, immunotherapy, hormonal therapy or other therapies) in the last 18 months of life and was eventually incorporated as a binary variable (yes/no).

#### Body image

Body image was measured by the validated Body Image Scale (BIS), with higher scores indicating a lower body image.^37^ The scale comprised of 10 questions and measured the perception of their body image in the past week, with responses standardized on a 0–30 scale.

#### Quality of life

Physical functioning and global quality of life were measured by the European Organization for Research and Treatment of Cancer Quality of Life Questionnaire C30 (EORTC QLQ-C30).^38^ For both physical functioning and global quality of life, responses were linearly transformed to a 0–100 score, with higher scores indicating better functioning/higher global quality of life.

### Statistical analysis

Descriptive statistics were used to summarize socio-demographic and clinical characteristics of patients and their partners. Time to death was categorized into cohorts of 3 months: 16–18, 13–15, 10–12, 7–9, 4–6, and 0–3 months before death to summarize the course of sexual desire, activity, satisfaction, and enjoyment over time. In addition, we calculated the differences in sexual health scores within couples at each time point, and reported the mean of these differences univariately to provide an overview of within-couple variation. Mixed-effects multivariable linear regression was performed to assess changes in sexual health over the last 18 months of the patient’s life for both patients and partners separately. The analysis among patients included time to death (continuous), age, sex, body image, treatment, comorbidities, physical functioning, primary tumor type, quality of life, and partner’s quality of life.^39^ The regression analyses of partners included patient’s time to death (continuous), treatment, primary tumor type and patients’ quality of life. Final regression analyses were performed to examine which aspects of sexual health were associated with patients’ quality of life, adjusted for variables that were significant in the primary analyses among patients. Statistical analyses were performed in STATA v17.0 and significance was set at *p* ⩽ 0.05.

## Results

**Figure 1.**
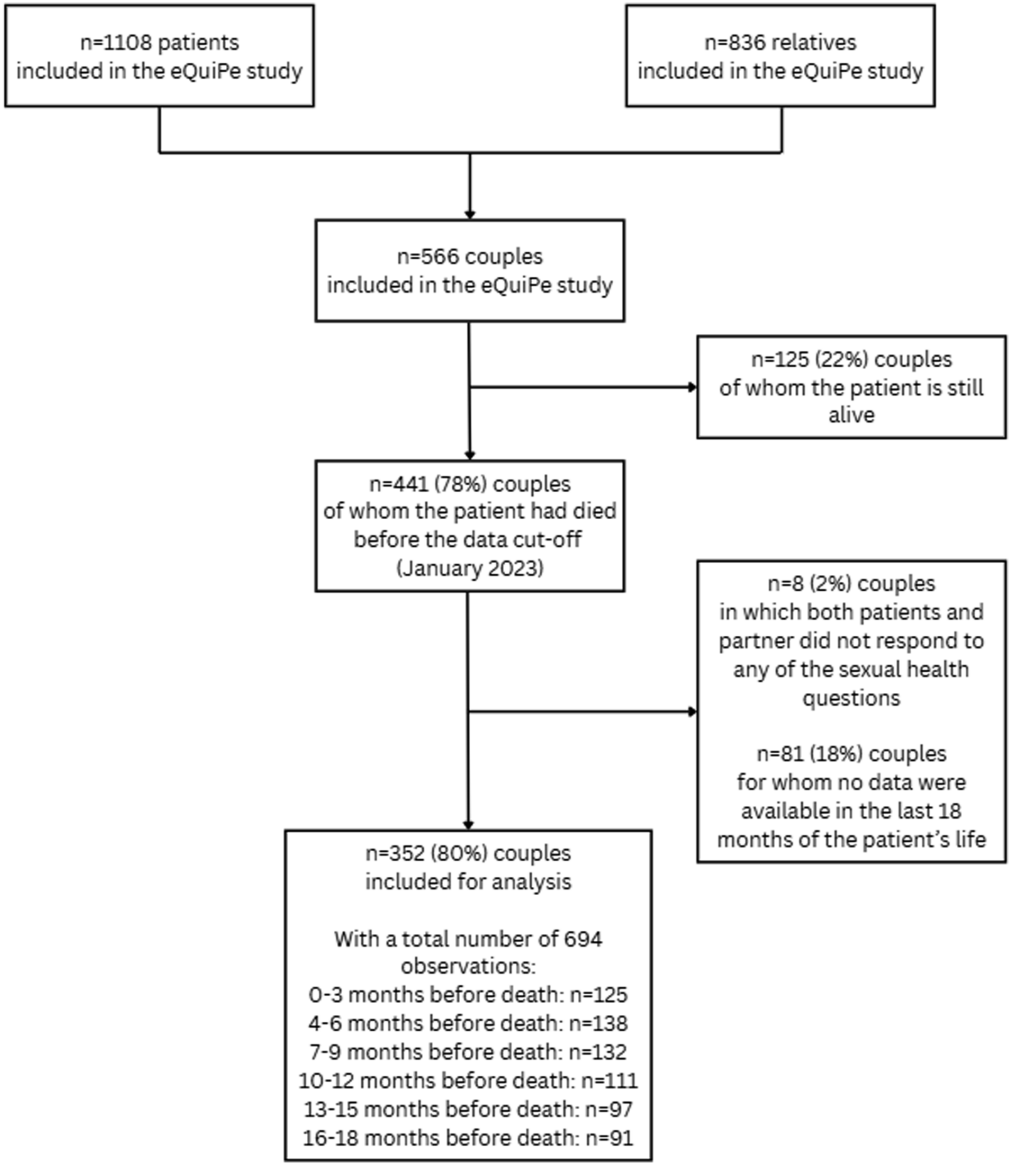
Flowchart of the study procedure.

In total, the eQuiPe study included 1108 patients and 836 relatives, with a response rate of respectively 65% and 71%. The eQuiPe study included 566 patient-partner couples and for this analysis, only couples in which the patients died before data cut-off (January 2023) and both the patients and partner completed at least one questionnaire in the last 18 months of the patient’s life were selected. In cases where neither the patients nor their partners responded to any of the sexual health questions, both the patient and the partner were excluded (*n* = 8). The final sample included 352 couples (Figure 1).

### Socio-demographic and clinical characteristics

Median number of questionnaires completed by each couple was 1, with a range of 1–6. Patients had a mean age of 66 years (SD 10) and 57% were male, and partners had a mean age of 65 years (SD 10; Table 1). The most common primary tumors were lung (26%), colorectal (20%) and breast (12%). There were four same-sex couples, and most couples (98%) had a relationship for more than 5 years and were living together at the moment of inclusion (99%).

**Table 1.** Socio-demographic and clinical characteristics of the study population.

Characteristics		Patients *N* = 352		Partners *N* = 352	
Age (years) Mean (SD), range		65.6 (9.5)	29–88	64.9 (10.0)	19–87
		*n*	%	*n*	%
Sex	Male	200	57	156	44
	Female	152	43	196	56
Religion	Yes	232	66	223	63
	No	118	34	128	37
Educational level^b^	Low	109	31	98	28
	Medium	137	39	159	45
	High	102	29	93	26
Relationship duration	≤ 5 years	6	2	6	2
	>5 years	346	98	346	98
Children^a^	Yes	282	80	300	85
	No	44	13	50	14
	Missing	26	7	2	1
Children living at home^a,b^	Yes	46	13	50	14
	No	236	67	248	70
	Missing	70	20	54	15
Primary tumor^b^	Lung	92	26	-	-
	Colorectal	72	20		
	Breast	41	12		
	Prostate	39	11		
	Other	107	30		
Received treatment in the last 18 months^b^	Yes	311	88	-	-
	No	40	11	-	-
Comorbidities^a^	Yes	220	63	-	-
	No	109	33		
	Missing	23	7	-	-

a Missing data were >5%.

b Percentages do not always add up to 100% because of missings.

### Sexual health in patients and partners in the end of life

**Figure 2.**
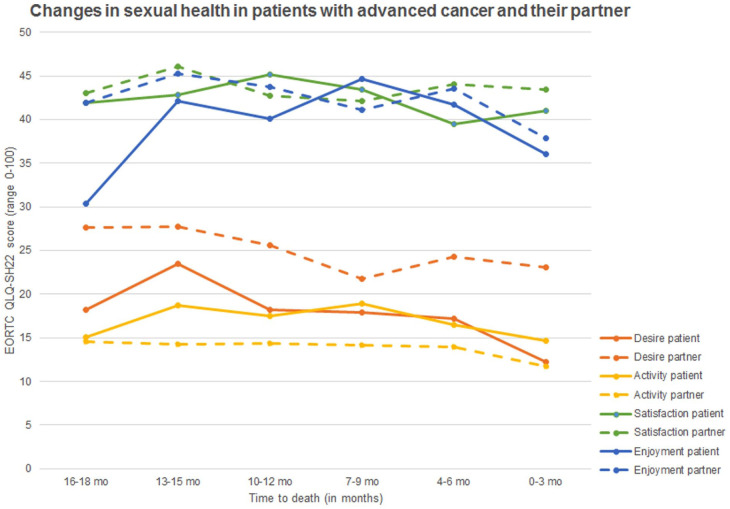
Sexual desire, activity, satisfaction, and enjoyment of patients with advanced cancer and their partner, in the last 18 months of life (n couples = 352).

Patients’ sexual activity (range 15–19) and satisfaction (range 40–45) were relatively stable in the last 18 months of life (Figure 2). Sexual desire decreased in the last 18 months, from 18 in 16–18 months before death to 13 in the last 3 months respectively. Enjoyment was 31 (SD 33) 16–18 months before death and 36 (SD 34) in the last 3 months of life. Mixed effect regression analysis confirmed that time to death was not associated with satisfaction, enjoyment or activity, only with a decrease in sexual desire (β 0.37, 95% CI 0.07–0.67; Table 2).

**Table 2.** Mixed-effects multivariable regression on the association between patient characteristics and sexual desire, activity, satisfaction, and enjoyment of patients.

	Desire *N* = 303			Activity *N* = 303			Satisfaction *N* = 303			Enjoyment *N* = 169		
	*Β*	95% CI	*p*-Value	β	95% CI	*p*-Value	β	95% CI	*p*-Value	β	95% CI	*p*-Value
Time to death	0.39	[0.08, 0.70]	**0.013**	−0.07	[−0.41, 0.27]	0.683	0.07	[−0.44, 0.57]	0.799	−0.42	[−1.15, 0.31]	0.256
Sex, men	Ref			.	.		.			.		
Women	−14.29	[−19.74, −8.83]	<**0.001**	−7.77	[−13.11, −2.43]	**0.004**	−0.27	[−8.11, 7.57]	0.946	−11.39	[−22.58, −0.19]	**0.046**
Age	−0.15	[−0.41, 0.12]	0.285	−0.20	[−0.46, 0.06]	0.136	−0.12	[−0.50, 0.26]	0.530	−0.34	[−0.88, 0.20]	0.217
Educational level, low	Ref			.			.			.		
Medium	0.37	[−5.16, 5.91]	0.895	−0.28	[−5.67, 5.10]	0.918	−0.01	[−7.92, 7.89]	0.998	11.15	[−0.37, 22.67]	0.058
High	−0.70	[−6.90, 5.51]	0.826	−1.61	[−7.87, 4.20]	0.552	1.63	[−7.22, 10.48]	0.718	12.42	[−0.65, 25.48]	0.063
Treatment, no	Ref			.			.			.		
Treatment, yes	1.03	[−2.93, 4.99]	0.609	−1.52	[−6.03, 2.81]	0.475	−1.76	[−8.39, 4.87]	0.603	0.17	[−9.64, 9.98]	0.973
Comorbidities, no	Ref			.			.			.		
Comorbidities, yes	−3.20	[−8.14, 1.75]	0.205	−1.52	[−6.33, 3.30]	0.537	3.87	[−3.21, 10.95]	0.284	3.46	[−6.55, 13.47]	0.498
Physical functioning	0.19	[0.11, 0.27]	<**0.001**	0.19	[0.10, 0.28]	<**0.001**	0.12	[−0.01, 0.25]	0.074	0.30	[0.10, 0.50]	**0.003**
Primary tumor, lung	Ref			.			.			.		
Colorectal	−2.30	[−9.31, 4.71]	0.520	−0.28	[−7.11, 6.55]	0.936	7.41	[−2.65, 17.46]	0.149	−6.68	[−20.44, 7.07]	0.341
Breast	1.28	[−7.27, 9.83]	0.769	6.66	[−1.65, 14.97]	0.116	18.38	[6.18, 30.57]	**0.003**	4.48	[−12.51, 21.47]	0.605
Prostate	−20.78	[−29.12, −12.44]	<**0.001**	−11.39	[−19.50, −3.28]	**0.006**	−3.29	[−15.21, 8.62]	0.588	−34.63	[−55.04, −14.22]	**0.001**
Other	−3.90	[−9.90, 2.11]	0.203	1.81	[−4.05, 7.67]	0.544	5.09	[−3.52, 13.70]	0.246	−2.23	[−14.40 9.93]	0.719
Body image	0.00	[−0.36, 0.37]	0.986	−0.01	[−0.40, 0.37]	0.952	−1.02	[−1.59, −0.45]	< **0.001**	−0.81	[−1.63, 0.02]	0.055
QoL partner	0.09	[−0.02, 0.20]	0.093	0.05	[−0.07, 0.16]	0.405	0.07	[−0.10, 0.23]	0.452	0.11	[−0.12, 0.35]	0.345

QoL: quality of life.

Bold formatting denotes statistical significance (*p*<0.05).

Partners sexual desire (range 22–28), activity (range 12–15), satisfaction (range 42–46), and enjoyment (range 39–46) remained relatively stable in the last 18 months of the patients’ life. Mixed effect regression analysis confirmed that time to death was not associated with sexual desire, activity, satisfaction, or enjoyment in partners (Table 3).

**Table 3.** Mixed-effects multivariable regression on the association between partner and patient characteristics and sexual desire, activity, satisfaction, and enjoyment of partners.

	Desire *N* = 298			Activity *N* = 296			Satisfaction *N* = 290			Enjoyment *N* = 154		
	*Β*	95% CI	*p*-Value	Β	95% CI	*p*-Value	β	95% CI	*p*-Value	β	95% CI	*p*-Value
Time to death^a^	0.19	[−0.12, 0.50]	0.225	0.16	[−0.14, 0.47]	0.294	−0.02	[−0.48, 0.44]	0.942	−0.09	[−0.76, 0.59]	0.806
Sex, men	Ref			.			.			.		
Women	−6.16	[−12.13, −0.19]	**0.043**	2.47	[−2.53, 7.47]	0.333	8.68	[0.20, 17.15]	0.045	3.80	[−6.35, 13.96]	0.463
Age	−0.69	[−0.99, −0.40]	<**0.001**	−0.27	[−0.51, −0.02]	**0.032**	0.16	[−0.25, 0.57]	0.434	−0.89	[−1.37, −0.41]	<**0.001**
Educational level, low	Ref			.			.			.		
Medium	−3.28	[−9.49, 2.94]	0.302	−0.36	[−5.59, 4.86]	0.892	4.54	[−4.31, 13.39]	0.315	4.56	[−6.26, 15.38]	0.408
High	2.22	[−4.87, 9.31]	0.540	1.85	[−4.12, 7.81]	0.544	−1.73	[−11.80, 8.33]	0.736	7.33	[−4.89, 19.54]	0.240
Treatment, no^a^	Ref			.			.			.		
Treatment, yes	1.47	[−2.57, 5.52]	0.476	−0.05	[−4.09, 3.98]	0.979	2.45	[−3.58, 8.48]	0.425	3.11	[−5.39, 11.60]	0.474
Comorbidities, no^a^	Ref			.			.			.		
Comorbidities, yes	−3.82	[−9.36, 1.73]	0.177	−2.95	[−7.58, 1.67]	0.211	0.59	[−7.20, 8.37]	0.882	0.22	[−9.23, 9.68]	0.963
Primary tumor, lung^a^	Ref			.			.			.		
Colorectal	−1.70	[−9.44, 6.03]	0.666	−1.42	[−7.91, 5.07]	0.669	−0.87	[−11.87, 10.13]	0.876	5.62	[−7.52, 18.75]	0.402
Breast	5.61	[−4.06, 15.27]	0.256	−2.70	[−10.86, 5.46]	0.517	−5.90	[−19.53, 7.73]	0.396	−14.15	[−30.47, 2.16]	0.089
Prostate	−1.95	[−11.08, 7.18]	0.675	−10.21	[−17.82, −2.60]	**0.009**	−2.37	[−15.18, 10.44]	0.717	−6.15	[−23.59, 11.28]	0.489
Other	0.69	[−6.11, 7.49]	0.843	−0.25	[−5.95, 5.45]	0.931	−0.13	[−9.80, 9.55]	0.980	4.72	[−6.69, 16.13]	0.417
QoL of patient^a^	0.09	[0.00, 0.18]	**0.045**	0.08	[−0.01, 0.16]	0.068	0.11	[−0.02, 0.24]	0.102	0.14	[−0.03, 0.32]	0.112

QoL: quality of life.

Bold formatting denotes statistical significance (*p*<0.05).

a Characteristics of the patient.

Sexual health scores within couples during the last 18 months of the patient’s life showed no substantial differences between patients and their partners. Patients reported slightly higher levels of sexual activity than their partners, with mean differences ranging from 0 to 7. In contrast, partners reported higher sexual desire scores compared to patients (mean difference range 4–11). Differences in sexual enjoyment (range 0–7) and satisfaction (range 0–5) varied between couples, with no consistent pattern of one group scoring higher than the other.

### Factors associated with sexual health in patients and partners at the end of life

Multivariable analyses showed that female patients had a steeper decline in sexual desire (β = −14.29, *p* < 0.05) and sexual activity (β = –7.77, *p* < 0.05) compared to male patients (Table 2). Better physical functioning was associated with higher scores on sexual desire (β = 0.19, *p* < 0.05), sexual activity (β = 0.19, *p* < 0.05) and sexual enjoyment (β = 0.30, *p* < 0.05; Table 2). Patients with a lower body image showed a significantly lower sexual satisfaction compared to those with a more positive body image (β = −1.02, *p* < 0.05).

In partners, female partners also reported significantly lower levels of sexual desire (β = −6.16, *p* < 0.05) compared to male partners and sexual desire (β −0.69, *p* < 0.05), sexual activity (β = −0.27, *p* < 0.05) and sexual enjoyment (β = −0.89, *p* < 0.05) decreased with older age (Table 3). Moreover, a better global quality of life of the patient was associated with higher sexual desire in partners (β = 0.09, *p* < 0.05).

### Association between sexual health and quality of life of patients over time

Sexual desire (β = 0.11, *p* < 0.05), sexual activity (β = 0.08, *p* < 0.05) and sexual satisfaction (β = 0.06, *p* < 0.05) were individually associated with higher global quality of life over time in patients (Table 4).

**Table 4.** Mixed-effects multivariable linear regression on the association between global quality of life and sexual desire, activity, satisfaction, and enjoyment in the last 18 months of life of patients with advanced cancer.^a^

Global QoL	*N*	*β*	95% CI	*p*-Value
Sexual desire	316	0.11	[0.04, 0.17]	**0.001**
Sexual activity	316	0.08	[0.02, 0.14]	**0.006**
Sexual satisfaction	316	0.06	[0.02, 0.10]	**0.007**
Sexual enjoyment	176	0.01	[−0.06, 0.07]	0.845

QoL: quality of life.

Bold formatting denotes statistical significance (*p*<0.05).

a This model is corrected for time to death, age, sex, physical functioning, body image, and primary tumor of the patient.

## Discussion

### Main findings

Couples confronted with advanced cancer reported low scores on sexual health at the end of life, which remained stable over time for both patients and their partners in most aspects. Only sexual desire in patients significantly further decreased during the end of life. Patients’ sexual health was associated with physical functioning, tumortype, and their partners’ age while partners’ sexual health was primarily associated with their own age. Moreover, sexual desire, activity, and satisfaction were significantly associated with global quality of life, indicating that these elements contribute to overall well-being in the last phase of life of patients with advanced cancer.

### What this study adds

The sexual health scores of patients with advanced cancer in this study are partially consistent with previous research on sexual health. Although normative data from Dutch cancer survivors indicating that 61% of individuals were not or minimally sexually active aligns with sexual activity scores in our study, normative data does show higher levels of sexual enjoyment in cancer survivors (72) compared to patients with advanced cancer included in our study (maximum of 44).^39^ Sexual enjoyment has not been widely explored before and, therefore, comparing our findings with existing literature is difficult. However, available results on sexual enjoyment in patients with curative cancer show either similar or higher scores compared to the levels of enjoyment in our study in patients with advanced cancer.^20,28,40^ Similarly, the mean sexual satisfaction of patients in our study (42) was similar to patients with recurrent or progressed cancer (39) reported by a recent cross-cultural field study,^41^ but lower than the normative population in Norway (53 in women and 58 in men).^42^ The lower levels of sexual enjoyment and sexual satisfaction in patients with advanced cancer suggest that patients may experience more problems with sexual health at the end of life than they do earlier in their (curative) cancer trajectory or compared to the cancer-free population.

Sexual health is often assumed to change at the end of life. However, in our study most aspects of sexual health remained stable during the end of life, except for patient’ sexual desire, this decreased in time toward death. Research on sexual health underpins this finding that for most patients the need for sexuality remains important, however there is a shift in sexuality toward less physicality, and sexuality is redefined toward a deeper emotional connection at the end of life.^15,16,29,43^ This shift in sexuality may (partly) explain why patients and partners in our study are relatively satisfied with their sex life, despite lower scores in sexual activity.

Our study also showed that some patients’ sexual health was more affected than others. Patients with prostate cancer showed a stronger decrease in sexual desire, activity, and enjoyment. Also, partners showed a decrease in sexual activity if patients were diagnosed with prostate cancer. Previous research concluded that patients with prostate cancer may experience sexual dysfunction due to androgen deprivation therapy and its impact on daily functioning, caused by the disease or treatment.^43–45^ This sexual dysfunction likely directly affects multiple aspects of sexual health, leading to lower sexual health scores, as seen in this study. Moreover, female patients had a greater decline in sexual desire and sexual activity, and female partners also showed lower scores in sexual desire, compared to men. This could be explained by previous studies that highlighted increased emotional distress in women, regardless of role (patient or partner),^46,47^ which may affect sexual health. Also, older partners showed a stronger decrease in sexual desire, activity, and enjoyment. An integrative review suggested that older patients with cancer are more likely to report decreased sexual health, in part due to barriers in seeking help caused by feelings of embarrassment and taboos.^48,49^ A higher age and decreased sexual activity are consistent with data from the general population in the United States.^50^ However, age did not affect sexual health in patients in our study, only of partners.

Decreased physical functioning of patients was also associated with a greater decline in multiple aspects of sexual health, including sexual desire, activity, and enjoyment. This is in line with a previous study in patients with metastatic cancer that showed that more than half of the patients reported that poor physical condition negatively affected their sex life.^7^ This can be explained by the fact that when patients experience worse physical functioning, it negatively affects their quality of life and therefore they may experience more barriers to engage in sexual interactions. Furthermore, most patients in this study received treatment, and it is known that the severity and intensity of treatment could, either directly or via other aspects of quality of life, affect the patients’ sexual health.^51^ We also showed that sexual desire, activity, and satisfaction were associated with a better overall quality of life, indicating that sexual health remains an important element of quality if life for patient at the end of life. This is in line with prior research, as patients reported that sexual health remained a priority, even at the end of life.^9,15^

In conclusion, these findings underpin the importance of discussing the topic of sexual health at the end of life. Multiple studies showed that patients with a life-threatening disease feel the need to discuss challenges in sexuality and intimacy^9,17^ and ⩾75% of oncology healthcare providers agreed that it is their responsibility to initiate such discussions.^19,50,51,52^ Nevertheless, healthcare providers often fail to initiate these discussions.^15,22,24,53,54^ Barriers to addressing sexual health include feelings of discomfort, inadequate training, time constraints, assumptions about elderly patients and sexuality, and limited understanding of intimate relationship dynamics in the context of metastatic cancer.^9,21,24,52,55,56^ Further research is essential to examine whether patients perceive decreased sexual health as a concern, as well as to assess their interest in discussing sexual health with their healthcare professional. The stepwise Permission Limited Information Specific Suggestions Intensive Therapy (PLISSIT) model is a tool that proved to be useful among healthcare providers.^57^ The PLISSIT model guides healthcare providers through a stepwise approach, beginning with permission to discuss sexual issues, followed by providing information, specific suggestions (e.g. recommending lubricants), and referring patients to more intensive therapy such as sex therapy or psychological support if needed. This structured approach helps create a supportive environment for patients to openly address their sexual health concerns.

### Limitations

Some limitations of the study need to be addressed. Patient recruitment was performed by the attending physician, a process that may have contributed to selection bias, this could lead to an overestimation of sexual health. Moreover, although the EORTC QLQ-SH22 has been validated in Dutch patients with advanced cancer,^41^ it is not widely used yet, hampering the comparison of results of this study to that of other studies. Also limited information regarding normative values are present of the sexual health items. Lastly, this study focused exclusively on couples to fill a gap in literature where the primary emphasis has been on patients only. However, a recent article has underscored that individuals who do not have a partner also deal with obstacles regarding intimacy and sexuality when dealing with terminal illness.^58^

## Conclusion

Sexual health in patients with advanced cancer and their partners remains relatively stable in the last 18 months of life, only sexual desire in patients decreases in time toward death. Patients with prostate cancer, female patients, and older partners are more likely to experience lower sexual health. Patients with a reduced physical functioning and/or prostate cancer are more likely to experience a greater decline in most aspects of sexual health and lower sexual health is associated with poorer quality of life. Maintaining or improving quality of life is of paramount importance for patients with incurable cancer and their partners. Therefore, it is important to address sexual health in couples facing terminal cancer, also within the context of palliative care. The use of short PROM’s, such as the PROMIS Sexual Functioning Short Form^59^ or the EORTC QLQ-SH22,^33^ to explore the need to discuss sexual health prior to meeting the healthcare professional could facilitate the initiation of such a discussion. These tools are validated for use in cancer populations and provide a concise yet effective assessment of sexual health.
